# Palmar and Plantar Tophi in Chronic Gout

**DOI:** 10.1007/s11606-026-10539-8

**Published:** 2026-05-26

**Authors:** Linh H. Tran, Josephine Du, Jessie Medina

**Affiliations:** University of California, Davis, School of Medicine, Sacramento, CA USA

**Figure 1:**
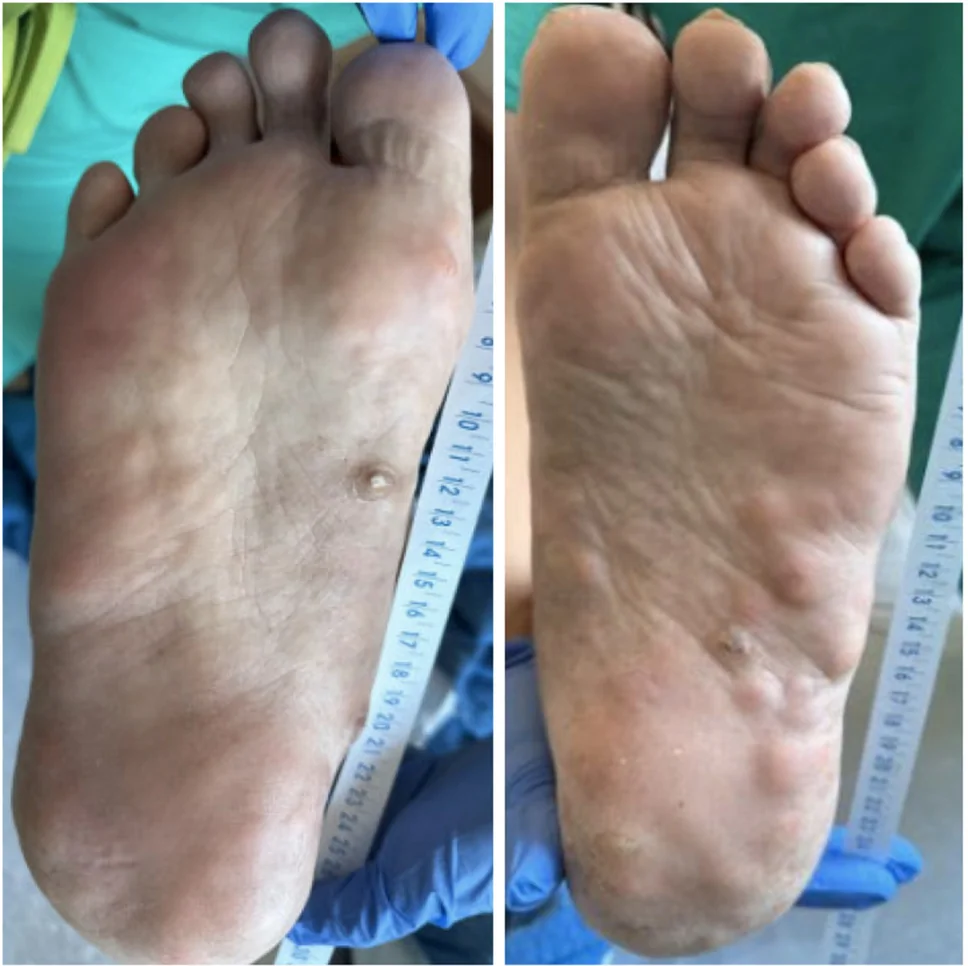
Multiple firm subcutaneous tophi over the plantar surfaces.

**Figure 2:**
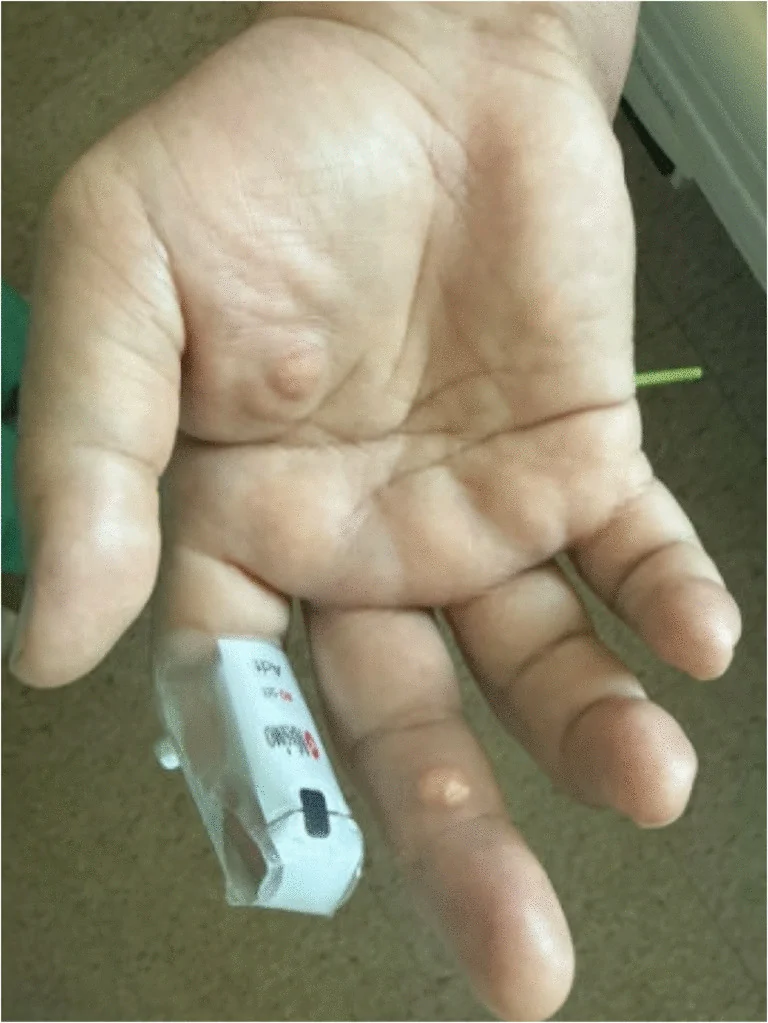
Subcutaneous tophi involving the palmar surfaces in chronic gout.

A 47-year-old man with diabetes, hypertension, and long-standing gout, off allopurinol, presented with low-grade fever and acute left knee pain. Examination revealed firm nodules over the interphalangeal joints, elbows, and palmar and plantar surfaces (Figs. 1 and 2). A serum uric acid of 11.1 mg/dL raised concern for presumed tophi. He received colchicine for a presumed acute gout flare with improvement and was counseled to resume allopurinol, titrated to serum urate < 6 mg/dL to reduce flares and promote tophus resorption^1,2^ after flare resolution.

Tophi typically occur in cooler sites such as the olecranon, Achilles tendon, and ears.^3^ Palmar and plantar involvement is exceptionally rare.^4,5^ Diagnosis at atypical sites can be confirmed by needle aspiration demonstrating negatively birefringent monosodium urate crystals on polarized microscopy.^3^ Plantar tophi may mimic warts or calluses,^5^ while palmar tophi may resemble calcinosis cutis, pyogenic pustules, or other nodular lesions,^4,6^ delaying diagnosis. These deposits are associated with chronic hyperuricemia and comorbidities such as diabetes and renal disease.^4,5,7,8^ Accurate diagnosis is critical, as these presentations can masquerade as infection or malignancy, prompting unnecessary procedures.^4^ For refractory tophaceous disease, pegloticase or febuxostat may be considered.^1,2^
